# Prognostic Factors in Tuberculosis Related Mortalities in Hospitalized Patients

**DOI:** 10.1155/2014/624671

**Published:** 2014-05-07

**Authors:** Ghazal Haque, Ashok Kumar, Fatima Saifuddin, Shafaq Ismail, Nadeem Rizvi, Shaista Ghazal, Sadhna Notani

**Affiliations:** Department of Chest Medicine, Jinnah Postgraduate Medical Centre, Rafiqui H J Shaheed Road, Karachi 75510, Pakistan

## Abstract

*Setting.* The study was undertaken at the Department of Pulmonology at a public, tertiary care centre in Karachi, Pakistan. *Objectives*. To evaluate factors concerned with in-hospital deaths in patients admitted with pulmonary tuberculosis (TB). *Design.* A retrospective case-control audit was performed for 120 patients hospitalised with pulmonary TB. Sixty of those discharged after treatment were compared to sixty who did not survive. Radiological findings, clinical indicators, and laboratory values were compared between the two groups to identify factors related to poor prognosis. *Results*. Factors concerned with in-hospital mortality listed late presentation of disease (*P* < 0.01), noncompliance to antituberculosis therapy (*P* < 0.01), smoking (*P* < 0.01), longer duration of illness prior to treatment (*P* < 0.01), and low body weight (*P* < 0.01). Most deaths occurred during the first week of admission (*P* < 0.01) indicating late referrals as significant. Immunocompromised status and multi-drug resistance were not implicated in higher mortality. *Conclusions*. Poor prognosis was associated with noncompliance to therapy resulting in longer duration of illness, late patient referrals to care centres, and development of complications. Early diagnosis, timely referrals, and monitored compliance may help reduce mortality. Adherence to a more radically effective treatment regimen is required to eliminate TB early during disease onset.

## 1. Introduction

Tuberculosis (TB) has been a declared worldwide health emergency since the last two decades. With an estimated 9 million new cases and 1.4 million deaths occurring annually, TB still stands to be a major global health risk despite rigorous efforts to contain its spread and implementation of effective treatment strategies [1].

Worldwide, Pakistan stands among the 22 countries that are most affected by TB. As of 2011, with an estimated population of 177 million, there were approximately 400,000 incident TB cases annually with about 59,000 deaths [2]. Every year, 15,000 new multi-drug resistant (MDR; resistant to isoniazid and rifampicin; first-line drugs) TB cases are reported in the country and an unprecedented number of cases remain unreported. Despite the availability of effective treatment regimens and clear regulations for their placement, active TB and its complications are a common cause of hospital admissions and TB related fatalities continue to persist. The reasons for these are widely distributed. While immunocompromised conditions [3, 4], disseminated disease [5], and environmental factors such as poor living and work conditions [6] are considered significant parameters, undernourishment [7] and anaemic [8] status, constant exposure to infected individuals, and disruption in treatment [9, 10] are also implicated.

To assess the factors associated with mortality, a holistic study is required to the weighing in all aspects of patient demographic, disease progression, treatment practices, and clinical and laboratory indicators. A retrospective case control study was carried out with patient data from February 2009 to March 2010 to analyse the implications of these elements on inpatient mortality.

## 2. Methods

The study was conducted at the department of Chest Medicine of the Jinnah Postgraduate Medical Centre (JPMC) in Karachi, Pakistan; a 1800-bed, public, tertiary care centre hosting 16 specialist facilities and 5 Intensive Care Units, admitting patients through outpatient clinics, and referrals. Patients included those with active TB in various treatment categories; requiring extensive treatment for complications of TB, multi-drug resistance, and TB with associated diseases.

A retrospective case control study was carried out on patients admitted to the hospital between February 2009 and March 2010 who were diagnosed with TB or its complications (Table 1). Patients' data were compared between two groups of 60 patients each: case patients who expired during hospitalization and controls who were discharged from the centre following treatment. Data were anonymously collected on a uniform, standardized questionnaire from filed records of patients who fulfilled the inclusion criteria. The hospital receives patients referred by primary and secondary care facilities from throughout the country, usually in advanced disease stages or with severe comorbid conditions; hence it was difficult to obtain follow up data on 68.3% controls who were unreachable after treatment. Compared cases and controls included those admitted to the centre in the same month of the year, with a positive diagnosis for TB, thus attempting to minimise bias since demographic, clinical, radiological, and other parameters were compared for their role in mortality. Inpatient records were examined for age and sex variations, clinical history and examination, chest radiographs obtained at the time of admission, and the subsequent treatment provided while hospitalised. The diagnosis for TB was made based on an initial evaluation by the admitting doctor and supported with clinical indicators for active TB in the history and examination: chronic (over months), persistent lowgrade fever with night sweats, productive cough resistant to antibiotic treatment, and weight loss and anorexia. Posteroanterior chest radiographs were studied to ascertain region of lung involvement and areas showing evidence of infiltration; cavitations or fibrosis was accounted as zones of involvement. Sputum smears tested for acid-fast bacilli (AFB) on Ziehl-Neelsen (ZN) stain and sputum cultures were used for confirmation wherever performed. Documentary proof of smears and cultures from a majority of the case patients was unavailable due to absent records and inadequate time between admission and death to perform fresh tests. However, evidence of previous ATT administration (complete or incomplete) was considered confirmatory for TB in such patients. Sputum cultures were received with all established cases of MDR-TB. TB was confirmed after a final assessment for the presence of one or more satisfactory diagnostic parameters by the resident postgraduate doctor.

Since the study was conducted at a dedicated pulmonology ward, cases of uniquely extrapulmonary TB were not available for inclusion. However, pulmonary TB cases that also exhibited extrapulmonary manifestations were discerned for significance. Further data recovered from patient files included duration of symptoms, comorbidities, history of TB contact and infection, smoking, disease and treatment status, weight at the time of admission, electrolyte and renal function panel, complete blood count, and blood gases.

It was difficult to obtain accurate histories of past TB infection and/or treatment due to unreliable patient accounts, improper documentation, and patient illiteracy. However, patients having received ATT with positive smears any time in the past were considered positive for a history of TB.

Those requiring category I ATT (new patients) were placed on a standard daily treatment [11] of isoniazid, rifampicin, pyrazinamide, and ethambutol for a two-month initial phase followed by a four-month continuation phase with isoniazid and rifampicin, while category II ATT (relapse or treatment default) added streptomycin to the initial phase and a five-month continuation phase with the addition of ethambutol. Patients with known drug resistances (category I failure or MDR-TB) were treated according to the reported drug sensitivity. Wherever possible, laboratory testing was performed via standardized procedures at the local lab conforming to ISO 9001:2008 certification.

Data collected were analyzed using PASW Statistics version 16. Categorical values were compared between cases and controls for risk estimation and odds ratios were reported with 95% confidence interval. Continuous values from laboratory data were analyzed through independent* t*-tests. A *P* value of <0.05 was considered significant.

## 3. Results

A total of 120 admitted patients who were diagnosed with TB were compared with each other: 60 case patients who did not survive hospitalization and 60 controls who were discharged after treatment (Table 2). Overall, there were 52 women (43.3%) and 68 men (56.6%). The male to female ratio was 1.14 : 1 and 1.5 : 1 among cases and controls, respectively, (*P* = 0.46). The mean age for controls was 32.9 years and 47 years for cases (*P* < 0.01). Most deaths occurred in the >40 age group with 35 fatalities of the 51 (68.6%, *P* < 0.01). On- admission body weights presented a mean of 48.8 kg for controls and 36.8 kg for cases (*P* < 0.01).

A past history of TB was found in 19 (31.6%) controls and 32 (53.3%) cases; 38 (63.3%) controls and 21 (35%) cases were primary diagnoses, while 3 controls and 7 cases could not provide the information (*P* < 0.01). History of close contact with a TB patient was reported in 18 (30%) patients from each group, while 42 (60%) controls and 28 (46.6%) case patients had no such contact. Data were unavailable for 14 of the case patients (*P* < 0.01).

Bilateral lung disease emerged as a significant factor, 31 cases (51.6%) showed bilateral disease and unilateral lung involvement was seen in 53 controls (88.3%, *P* < 0.01).

Sputum smears were positive for AFB in 39 (65%) controls, 17 (25%) tested negative, and smears were unavailable for 4 controls. Smears were reported on only 25 case patients out of which 15 were AFB-positive. MDR-TB was reported in 4 controls and 8 cases (*P* < 0.01). On admission, 56 (82.4%) controls and only 12 (17.6%) cases were taking ATT (*P* < 0.01). These included 46 controls and 6 cases on category I, 6 controls and 1 case on category II, and 4 controls and 5 cases were on MDR treatment (*P* < 0.01) [11].

Pulmonary TB limited to lung parenchyma was present in 39 (65%) case patients and only 16 (26.6%) controls. Both pulmonary and extrapulmonary TB were found in 44 (73.3%) controls and 21 (35%) cases (*P* < 0.01). Complications of TB were seen on admission in some patients including high risk factors such as type II respiratory failure, pulmonary fibrosis, and dissemination (Table 3). Other recorded conditions included systemic arterial hypertension (12 cases and 5 controls) and diabetes (8 cases and 3 controls). Two patients had asthma, 3 had COPD, 2 had hepatitis C, and 1 had carcinoma of the lung; all of these were case patients. Save for 6 case patients, test for human immunodeficiency virus (HIV) was performed on all participants and only 1 case patient tested positive (*P* = 0.02).

A positive history of tobacco use was found in 11 (18.3%) controls and 32 (53.3%) cases (*P* < 0.01). Mean pack years for controls were 4.5 and 13.9 for cases (*P* < 0.01). A large number of smokers (60.5%) contracted pulmonary TB alone while 62.3% (48 of 77) of nonsmokers had both pulmonary and extrapulmonary TB (*P* = 0.01). Passive smoking was reported in 18 (30%) controls and 44 (73.3%) cases and was associated with high mortality (*P* < 0.01). A comparative profile of smokers and nonsmokers is given (Table 4).

The mean duration of symptoms among controls was 19 months and for cases was 5 years (*P* < 0.01). Shorter duration of symptoms before admission was associated with better chances of survival. In the group of patients with less than 6 months duration of symptoms, 37 of 53 (69.8%) survived treatment (*P* < 0.01).

**Figure 1:**
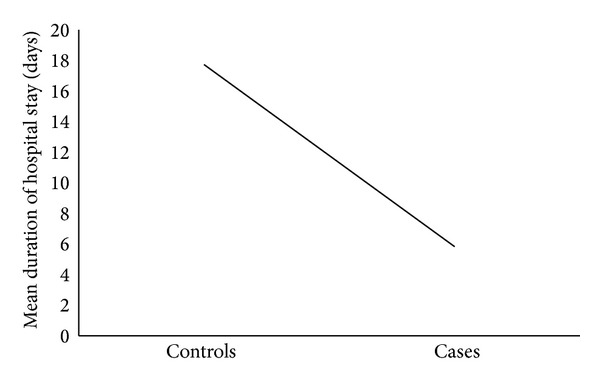
Relationship between mean number of hospitalization days and patient outcome.

A majority of deaths (78.3% of cases) occurred in the first week (*P* < 0.01), indicating that most patients reached the centre at terminal stages of disease (Figure 1). Mean hospital stay for controls was 17.73 days (range 3–74 days) while that for case patients was 5.83 days (range 0–29 days, *P* < 0.01).

At the time of admission raised leukocytes, neutrophilia, lymphocytopaenia, and low serum protein were found significantly related to mortality (Table 1).

## 4. Discussion

Tuberculosis stands as the second leading cause of death worldwide despite the enforcement of comprehensive prevention, detection, and treatment measures. Although the mortality rate of TB has reduced by 41% in the last two decades, many countries with a high burden of TB still have a long way to go in disease eradication [1]. Incidences of HIV coinfection [12], MDR-TB, and extensively drug-resistant TB (XDR-TB; resistance to at least isoniazid and rifampicin, any fluoroquinolone, and to injectable amikacin, capreomycin, or kanamycin) have raised concerns in many regional treatment strategies [13]. Pakistan ranks among countries with both a high burden of TB and MDR-TB and encounters a high mortality rate from TB [2]. Efforts have been made in the past to analyze the causes of high mortality in TB patients and for their poor prognosis [14–18], especially in hospitalized patients [12, 19, 20]. HIV and other immunocompromised status [21], MDR-TB [22], late referrals [23], and increased age of patients [24] have been implicated, but these factors can vary regionally. Our research aimed to analyze these factors from a local perspective to examine the causes of poor prognosis in hospitalized TB patients in Karachi.

While studies [4–6, 12, 15–19, 21] from most countries have reported concomitant HIV to be an important factor in TB mortality, Pakistan fortunately escapes the list of countries with a high HIV burden and thus, it is not considered a major risk factor for high mortality with pulmonary TB [25, 26]. In the course of this study, HIV was detected in only one case patient and hence no significant association could be established.

Patients belonging to the >40-age group were more at risk compared to younger subjects. The combination of advanced age, deferred treatment, and frequent complications was a marker of poor prognosis. The male to female ratio was very slightly higher for cases. Low body weight was an important factor compounded by association with undernourishment and anaemia. Although no statistical significance resulted between mortality and low haemoglobin levels, clinical anaemia was observed in over half of the cases and under half of the controls. Moreover, a majority of the patients had lymphocytopenia and hypoproteinaemia, suggesting a generally malnourished, disease-susceptible patient outlook [7].

With a high burden environment for TB and scarce resources, sputum smears were observed when a clinical and radiological diagnosis was uncertain. Although nearly 3/4 of patients receiving ATT had positive smears, there was also a small number of retreated patients who had to be placed on category II treatment [27]. However, over 90% of those who were not taking ATT died. This can be accounted to illiteracy, low awareness regarding ATT, nonadherence to directly observed treatment (DOT) programmes, and low level of social support. Most patients hailed from low-income, rural settings and cited lack of infrastructural support as a reason for noncompliance [28]. MDR-TB has presented treatment challenges for countries with a high burden of MDR-TB [29]. We received 12 patients with established drug-resistant isolates on culture and only 4 of them survived. Notably, failure of getting followup exams and sputum smears likely results in a good number of relapse and MDR cases going undetected in our setting.

Poor record keeping and widespread reliance on nondigital filing systems presented difficulties in obtaining accurate treatment and disease histories and patient accounts were largely unreliable. However, nearly half of the patients in the study were new TB cases while a majority of the other half was cases of interrupted treatment or default. A surprisingly long duration of symptoms was seen in all the patients. This was attributed to the patients' neglect to report and seek medical assistance early on during the disease due to lack of awareness, resources, and social stigma. Long-established cultural practices among patients of visiting unqualified persons and quacks resulted in delayed detection and treatment [10].

Indigenous pulmonary TB and bilateral lung involvement were poor prognostic factors for hospitalized patients, usually associated with the development of complications. Conversely, patients with TB-involving sites other than the lung parenchyma fared better. Long-term complications of TB significantly raised the risk of mortality for inpatients [30, 31] at times requiring intensive care or ventilation support [32, 33]. Long-term sequelae of TB such as pulmonary fibrosis, type II respiratory failure, and disseminated disease were indicators of poor prognosis. Most deaths occurred within the first week of admission implicating late referrals and subsequent complications since these patients could not benefit from further treatment.

Various studies [34–36] have reported a strong association between tobacco use and increased risk of TB incidence and mortality. Passive smoking too is considered a health risk [37], yet Pakistan remains in the top five countries in the world with both a high burden of TB and tobacco usage. Our study revealed 75% mortality from TB among smokers. Moreover, these patients exhibited lower body weight, bilateral lung involvement, increased risk of infections, and developing life-threatening complications when compared to their nonsmoking counterparts.

## 5. Conclusions

The conclusions drawn from this study indicate delayed and interrupted treatment, failure to refer complications, nonadherence to treatment programmes, and tobacco usage as risk factors for increased TB mortality in hospitalized patients. We recommend stringent implementation of standardized treatment regimens [11] in tandem with local restrictions in their practice. Concrete statistical data on the incidence of these factors needs to be meticulously obtained to outline remedial lines of action. Awareness programmes and stricter regulations for TB and antitobacco use are pertinent to reduction in TB incidence. Simple corrective measures such as better record keeping, educating patients and caregivers regarding treatment, and creating awareness regarding TB and tobacco use can go a long way in preventing high mortality and curtailing this epidemic.

## Figures and Tables

**Table 1:** Diagnostic, inclusion, and exclusion criteria for cases and controls*.

Parameters for TB diagnosis	
Positive sputum smear and/or culture for AFB	
CXR with evidence of cavitations, infiltration, and/or fibrosis	
Clinical indicators of TB, chronic persistent: low-grade fever with night sweats, productive cough, weight loss, and anorexia	
Inclusion criteria	
Fulfilling two or more diagnostic parameters for pulmonary TB	
Patients admitted within the same month of the year	
Exclusion criteria	
No clear evidence of active pulmonary TB	
Exclusively extrapulmonary TB	
Cause of death other than TB (for cases)	

*TB: tuberculosis; AFB: acid-fast bacilli; CXR: chest X-ray.

**Table 2:** Patient particulars on admission with odds ratio*.

Factor	Cases	Controls	*P*	OR (95% CI)
Female sex, *N* (%)	28 (46.6)	24 (40)	0.46	0.76 (0.36–1.57)
Age, mean years (range)	47 (14–95)	32.97 (15–75)	0.04	n/a
Weight, mean kg (range)	36.82 (28–65)	48.83 (25–81)	<0.01	n/a
Smokers, *N* (%)	32 (53.3)	11 (18.3)	<0.01	0.19 (0.08–0.44)
Past TB history, *N*(%)	32 (53.3)	19 (31.6)	0.04	0.32 (0.15–0.71)
Pulmonary TB, *N* (%)	39 (65)	16 (26.6)	<0.01	0.19 (0.09–0.42)
Bilateral lung involvement, *N* (%)	31 (51.6)	7 (11.6)	<0.01	8.09 (3.17–20.6)
Not taking ATT, *N* (%)	48 (80)	04 (6.6)	<0.01	56 (16.9–185.0)
Smear positive, *N*	15/25	39/56	<0.01	n/a
HIV positive, *N* (%)	1/54	00	0.02	n/a
TLC ×10^9^/L, mean	12.91	8.27	<0.01	n/a
Neutrophils, mean %	82.60	77.07	0.02	n/a
Lymphocytes, mean %	12.49	18.27	<0.01	n/a
Serum protein U/L, mean	5.01	6.48	<0.01	n/a

*OR: odds ratio; CI: confidence interval; TB: tuberculosis; ATT: anti-tuberculosis treatment; HIV: human immunodeficiency virus; TLC: total leukocyte count; n/a: not applicable.

**Table 3:** Complications and variations of TB, *N* (%).

	Cases	Controls	P*
Complication of TB			
Type II respiratory failure	18 (30)	1 (1.6)	<0.01
Pulmonary fibrosis	19 (31.6)	8 (13.3)	0.01
Disseminated TB	11 (18.3)	3 (5)	0.02
Post-TB bronchiectasis	12 (20)	7 (11.6)	0.21
Variation of TB			
Pleural effusion	9 (15)	27 (45)	<0.01
Hydropneumothorax	2 (3.3)	12 (20)	<0.01
Miliary TB	1 (1.6)	3 (5)	<0.01
Empyema	1 (1.6)	2 (3.3)	<0.01
Lymph node TB	1 (1.6)	0 (0)	<0.01
Multiple sites	7 (11.6)	0 (0)	<0.01

*P value for variations of TB is within observed instances of extrapulmonary TB.

**Table 4:** Patient profile: smokers versus nonsmokers*.

Feature	Smoker	Nonsmoker	*P *	OR (95% CI)
Patients, *N* (%)	43/120 (35.8)	77/120 (64.1)	<0.01	n/a
Cases, *N* (%)	32/43 (74.4)	28/77 (36.4)	<0.01	2.04 (1.45–2.88)
Controls, *N* (%)	11/43 (25.6)	49/77 (63.6)	<0.01	0.40 (0.23–0.68)
Age, mean years	48.49	35.23	<0.01	n/a
Weight, mean kg	41.74	43.43	0.46	n/a
Male sex, *N* (%)	34/43 (79.1)	34/77 (50)	<0.01	0.20 (0.08–0.49)
Bilateral lung involvement, *N* (%)	18/43 (41.9)	20/77 (26)	0.07	0.48 (0.22–1.07)
Pulmonary fibrosis, *N* (%)	12/43 (27.9)	15/77 (19.5)	0.28	1.60 (0.66–3.83)
Type II respiratory failure, *N* (%)	10/43 (23.3)	9/77 (11.7)	0.09	2.29 (0.84–6.17)
Duration of hospital stay, mean days	8.8	13.4	0.02	n/a
TLC ×10^9^/L, mean	11.7	9.9	0.12	n/a
Neutrophils, mean %	84.0	78.1	0.02	n/a
Serum protein U/L, mean	5.09	6.11	<0.01	n/a

*OR: odds ratio; CI: confidence interval; TLC: total leukocyte count; n/a: not applicable.

## References

[1] World Health Organization (2012). *Global Tuberculosis Report 2012*.

[2] World Health Organization (2012). *Global Tuberculosis Report 2012 Annex 2: Country Profiles*.

[3] World Health Organization TB/HIV facts 2012-13. http://www.who.int/hiv/topics/tb/tbhiv_facts_2013/en/index.html.

[4] Kivihya-Ndugga LE, Ochola JJ, Otieno G, Muthami LN, Gathua S (1994). Clinical and immunological markers in Kenyan pulmonary tuberculosis patients with and without HIV-1. *East African Medical Journal*.

[5] Burton NT, Forson A, Lurie MN, Kudzawu S, Kwarteng E, Kwara A (2011). Factors associated with mortality and default among patients with tuberculosis attending a teaching hospital clinic in Accra, Ghana. *Transactions of the Royal Society of Tropical Medicine and Hygiene*.

[6] Lönnroth K, Jaramillo E, Williams BG, Dye C, Raviglione M (2009). Drivers of tuberculosis epidemics: the role of risk factors and social determinants. *Social Science and Medicine*.

[7] Kim H-J, Lee C-H, Shin S (2010). The impact of nutritional deficit on mortality of in-patients with pulmonary tuberculosis. *International Journal of Tuberculosis and Lung Disease*.

[8] Isanaka S, Aboud S, Mugusi F (2012). Iron status predicts treatment failure and mortality in tuberculosis patients: a prospective cohort study from Dar es Salaam, Tanzania. *PLoS ONE*.

[9] Kliiman K, Altraja A (2010). Predictors and mortality associated with treatment default in pulmonary tuberculosis. *International Journal of Tuberculosis and Lung Disease*.

[10] Finlay A, Lancaster J, Holtz TH, Weyer K, Miranda A, Van Der Walt M (2012). Patient- and provider-level risk factors associated with default from tuberculosis treatment, South Africa, 2002: a case-control study. *BMC Public Health*.

[11] World Health Organization (2010). *Treatment of Tuberculosis: Guidelines—4th Edition. WHO Report 2010*.

[12] Sacks LV, Pendle S (1998). Factors related to in-hospital deaths in patients with tuberculosis. *Archives of Internal Medicine*.

[13] Glaziou P, Floyd K, Korenromp EL (2011). Lives saved by tuberculosis control and prospects for achieving the 2015 global target for reducing tuberculosis mortality. *Bulletin of the World Health Organization*.

[14] Tiemersma EW, van der Werf MJ, Borgdorff MW, Williams BG, Nagelkerke NJD (2011). Natural history of tuberculosis: duration and fatality of untreated pulmonary tuberculosis in HIV negative patients: a systematic review. *PLoS ONE*.

[15] van't Hoog AH, Williamson J, Sewe M (2012). Risk factors for excess mortality and death in adults with tuberculosis in Western Kenya. *The International Journal of Tuberculosis and Lung Disease*.

[16] Waitt CJ, Squire SB (2011). A systematic review of risk factors for death in adults during and after tuberculosis treatment. *International Journal of Tuberculosis and Lung Disease*.

[17] Selig L, Belo MTCT, Teixeira EG (2003). The study of tuberculosis-attributed deaths as a tool for disease control planning in Rio de Janeiro, Brazil. *International Journal of Tuberculosis and Lung Disease*.

[18] Selig L, Guedes R, Kritski A (2009). Uses of tuberculosis mortality surveillance to identify programme errors and improve database reporting. *International Journal of Tuberculosis and Lung Disease*.

[19] Alavi-Naini R, Moghtaderi A, Metanat M, Mohammadi M, Zabetian M (2013). Factors associated with mortality in tuberculosis patients.. *Journal of Research in Medical Sciences*.

[20] Horita N, Miyazawa N, Yoshiyama T (2013). Development and validation of a tuberculosis prognostic score for smear-positive in-patients in Japan. *The International Journal of Tuberculosis and Lung Disease*.

[21] Nahid P, Jarlsberg LG, Rudoy I (2011). Factors associated with mortality in patients with drug-susceptible pulmonary tuberculosis. *BMC Infectious Diseases*.

[22] Donald PR, Van Helden PD (2009). The global burden of tuberculosis—combating drug resistance in difficult times. *New England Journal of Medicine*.

[23] Liu Y-C, Lin H-H, Chen Y-S (2010). Reduced health provider delay and tuberculosis mortality due to an improved hospital programme. *International Journal of Tuberculosis and Lung Disease*.

[24] Feng J-Y, Su W-J, Chiu Y-C (2011). Initial presentations predict mortality in pulmonary tuberculosis patients—a prospective observational study. *PLoS ONE*.

[25] Syed ZA, Khan MI, Aslam M, Taseer IH (2003). Frequency of HIV infection in patients of pulmonary tuberculosis and various cancers at Nishtar Hospital Multan. *Pakistan Journal of Medical Research*.

[26] Chaudhry MK, Syed ZA, Younus M (2009). Prevalence of Human Immunodeficiency Virus infection in patients with pulmonary tuberculosis. *Pakistan Journal of Chest Medicine*.

[27] Marx FM, Dunbar R, Enarson DA, Beyers N (2013). The rate of sputum smear-positive tuberculosis after treatment default in a high-burden setting: a retrospective cohort study. *PLoS ONE*.

[28] Deivanayagam CN (2006). The challenges of tuberculosis. *The Indian Journal of Chest Diseases & Allied Sciences*.

[29] Nathanson E, Nunn P, Uplekar M (2010). MDR tuberculosis—critical steps for prevention and control. *New England Journal of Medicine*.

[30] Ryu YJ, Lee JH, Chun E-M, Chang JH, Shim SS (2011). Clinical outcomes and prognostic factors in patients with tuberculous destroyed lung. *International Journal of Tuberculosis and Lung Disease*.

[31] Kim HY, Song K-S, Goo JM, Lee JS, Lee KS, Lim T-H (2001). Thoracic sequelae and complications of tuberculosis. *Radiographics*.

[32] Zahar J-R, Azoulay E, Klement E (2001). Delayed treatment contributes to mortality in ICU patients with severe active pulmonary tuberculosis and acute respiratory failure. *Intensive Care Medicine*.

[33] Silva DR, Menegotto DM, Schulz LF, Gazzana MB, Dalcin PTR (2010). Mortality among patients with tuberculosis requiring intensive care: a retrospective cohort study. *BMC Infectious Diseases*.

[34] World Health Organization and International Union Against Tuberculosis and Lung Disease (2007). *A WHO/The Union Monograph on TB and Tobacco Control: Joining Efforts to Control Two Related Global Epidemics*.

[35] Underner M, Perriot J (2012). Smoking and tuberculosis. *Presse Medicale*.

[36] Lin H-H, Ezzati M, Chang H-Y, Murray M (2009). Association between tobacco smoking and active tuberculosis in Taiwan: Prospective cohort study. *The American Journal of Respiratory and Critical Care Medicine*.

[37] Leung CC, Lam TH, Ho KS (2010). Passive smoking and tuberculosis. *Archives of Internal Medicine*.

